# Predicting regional gray swans via translocation: AI weather models and Dubai’s unprecedented 2024 rainfall

**DOI:** 10.1126/sciadv.ady9406

**Published:** 2026-10-07

**Authors:** Qiang Sun, Pedram Hassanzadeh, Tiffany Shaw, Hamid A. Pahlavan, Adam Marchakitus

**Affiliations:** ^1^ Department of the Geophysical Sciences, University of Chicago, Chicago, IL 60637, USA.; ^2^ State Key Laboratory of Severe Weather Meteorological Science and Technology, Nanjing University, Nanjing 210023, China.; ^3^ Committee on Computational and Applied Mathematics, University of Chicago, Chicago, IL 60637, USA.; ^4^ NorthWest Research Associates, Boulder, CO 80301, USA.

## Abstract

Artificial intelligence (AI) models have transformed weather forecasting, but their skill for unprecedented weather extremes is unclear. Here, we analyze GraphCast, AIFS, and FuXi forecasts of the unprecedented 2024 Dubai storm, which had twice the training set’s highest rainfall in that region. GraphCast and AIFS accurately forecast this event up to 8 days ahead. FuXi forecasts the event but underestimates the rainfall. Fine-tuning and receptive field analyses suggest that these models’ success stems from “translocation”: learning from comparable/stronger dynamically similar events in other regions during training. Evidence of “extrapolation” (learning from weaker events) is not found. Even events within the global distribution’s tail are poorly forecasted, which is not only due to data imbalance (generalization error) but also spectral bias (optimization error). These findings demonstrate the potential of AI models to forecast “regional” gray swans and the opportunity to improve them through understanding the mechanisms behind their successes/limitations.

## INTRODUCTION

Artificial intelligence (AI) weather models such as FourCastNet (*1*), Pangu (*2*), GraphCast (*3*), FuXi (*4*), AIFS (*5*), NeuralGCM (*6*), and GenCast (*7*) have demonstrated remarkable skill in forecasting global weather patterns on short- to medium-range timescales. These AI models consistently match or even surpass the performance of state-of-the-art physics-based numerical weather prediction (NWP) models. The key advantages of these AI weather models—which include higher accuracy, orders-of-magnitude faster forecasting speed, and substantially lower computational cost (*1*, *2*, *8*–*11*)—have captured the attention of the research community and are beginning to gain traction within operational weather centers (*5*, *12*–*16*) and organizations focused on improving global access to weather forecasts (*17*–*19*).

Despite the overall success of these AI models in short- and medium-range weather forecasting based on common metrics, there are concerns about their ability to reliably forecast the rarest yet most extreme weather events, particularly the so-called “gray swans” (*3*, *20*–*23*). First used in climate science by Lin and Emanuel (*24*), a gray swan refers to a rare extreme weather event that is physically possible but, due to its long return period, has not been seen in the available historical record. Recently, Sun *et al.* (*25*) used this term in the context of AI weather/climate modeling for physically possible extreme events that are so strong and rare that they are effectively out of distribution (OOD). We will define (regional versus global) gray swans and OOD more rigorously in the “Factors contributing to the success of AI forecasts for Dubai’s unprecedented rainfall event” section; briefly, OOD here means that the intensity of at least one input or output variable of the AI model exceeds any value seen in the training set in that region (grid point).

Forecasting gray swan events accurately requires AI models to generalize OOD (beyond their training data distribution), a challenge that potentially necessitates learning underlying physical processes rather than merely recognizing patterns. The AI models’ ability to forecast OOD events, such as gray swans, poses a significant test of their generalization capacity. This question also has major social implications, given the large impact of these strongest weather events on the one hand and the rapid rise of AI-driven operational weather forecasting on the other (*18*–*20*, *26*–*28*).

Several case studies have begun to explore the ability of AI models to forecast individual extreme events (*29*–*33*). However, further analyses are needed to quantify the extent to which these test cases were beyond the training distribution, both regionally and globally. Moreover, much of the existing literature focuses on evaluating the models’ forecast skills without delving into the mechanisms that underpin their success or failure. However, understanding why different models perform differently in forecasting certain extreme events is critical for further advancing AI weather forecasting systems.

For example, the error of deep learning models can be decomposed into three sources: approximation, generalization, and optimization (*34*, *35*). The difficulty in learning extreme weather events is often attributed to their rarity, the classic “data imbalance” problem in statistical learning, a form of generalization error (*36*, *37*). Gray swans represent the asymptotic limit of the data imbalance problem. However, the large amplitudes of these events also make them prone to what is known as the blurring (or double penalty) problem in the AI for climate literature (*21*, *38*), which is the classic “spectral bias” in deep learning (*39*). Theoretical work has shown that spectral bias, i.e., the challenges of neural networks in learning small scales (high wave number), is an optimization error (*40*, *41*). Given that errors from data imbalance and spectral bias have different roots and thus different potential remedies, it is critical to understand their roles in investigations of AI weather models’ performance for extreme events and, in particular, for gray swans.

With these questions in mind, here, we examine the medium-range forecast skill of three state-of-the-art AI weather models, GraphCast, AIFS, and FuXi, in detail for the April 2024 extreme rainfall in Dubai and a few other extreme rainfall episodes in the Northern Hemisphere. We focus on the Dubai event for two reasons. First, as shown later, this record-breaking event had nearly twice the maximum rainfall previously observed in this region in the training datasets of these models, making it an ideal OOD event. In addition to examining the effects of data imbalance and spectral bias on the AI-driven forecasts of this event, we investigate these AI models’ ability to learn from the extreme events occurring in other regions through a combination of controlled fine-tuning experiments and diagnostic analysis of the effective receptive field (ERF) of the neural network. These analyses provide insight into the strengths and weaknesses of these models in forecasting OOD extreme events, thus informing future developments in AI-based weather prediction systems. Second, while recent advances have demonstrated the skill of AI models in nowcasting (*42*–*44*) or short-term forecasting (*5*, *7*, *21*) of rainfall, their performance for extreme rainfall events, especially gray swans, remains unexplored.

This gap is critical, as these events are known to be difficult to predict with traditional NWP models and often have severe societal impacts. Dubai’s April 2024 event serves as an example: As an arid region unprepared for these extremes, the city experienced widespread flooding and infrastructure damage, with estimated insurance losses of approximately US$3 billion and five reported fatalities (*45*). This highlights the urgent need for improved forecasting capabilities and the rapid public dissemination of reliable early warnings. The possibility of AI models to skillfully predict extreme rainfall several days in advance, as examined in this study, combined with their computational efficiency and public availability, offers a promising new pathway for delivering early warnings through multimodel, large-ensemble forecasts (*17*, *28*).

## RESULTS

### Skill of AI models for Dubai’s unprecedented rainfall event

Dubai’s extreme rainfall event, which began around 15 April 2024, is evident in the ERA5 reanalysis data, with a peak 12-hour accumulated value of 60 mm on 16 April, even after averaging over a 100 km–by–100 km box centered around the city (Fig. 1A). This value is almost twice the maximum Dubai precipitation in the training set of the AI models (33 mm), making this an OOD event (see the histogram in Fig. 2A). We find this event’s daily rainfall to have a return period of ∼150 years (see Materials and Methods). However, with a 5-day lead time, all three models, GraphCast, AIFS, and FuXi, which provide precipitation forecasts, accurately capture the timing of the event, with an error margin of one or two 6-hourly time steps. Notably, GraphCast’s and AIFS’s 5-day forecasts predict a maximum precipitation value that is over 50 mm (on 16 April), closely matching that of ERA5 and far exceeding the maximum value of the training data for the Dubai region (Fig. 1A). Although both GraphCast and AIFS slightly underestimate the total accumulated rainfall over the event period (15 to 17 April) relative to the ground “truth” in the ERA5 statistical domain (by ∼10 and ∼15%, respectively), their ability to forecast the timing, location (Fig. 1, C and D, and fig. S2), and the overall amplitude of such an unprecedented event is somewhat expected. This finding might appear to suggest GraphCast’s and AIFS’s ability to generalize OOD. Note that even the 8-day forecast skill of GraphCast is reasonable, capturing the circulation and rainfall patterns (fig. S1) and producing peak rainfall over 40 mm around Dubai on 16 April. AIFS shows comparable skill in predicting this event at 7- to 8-day lead time (fig. S2).

**Fig. 1.**
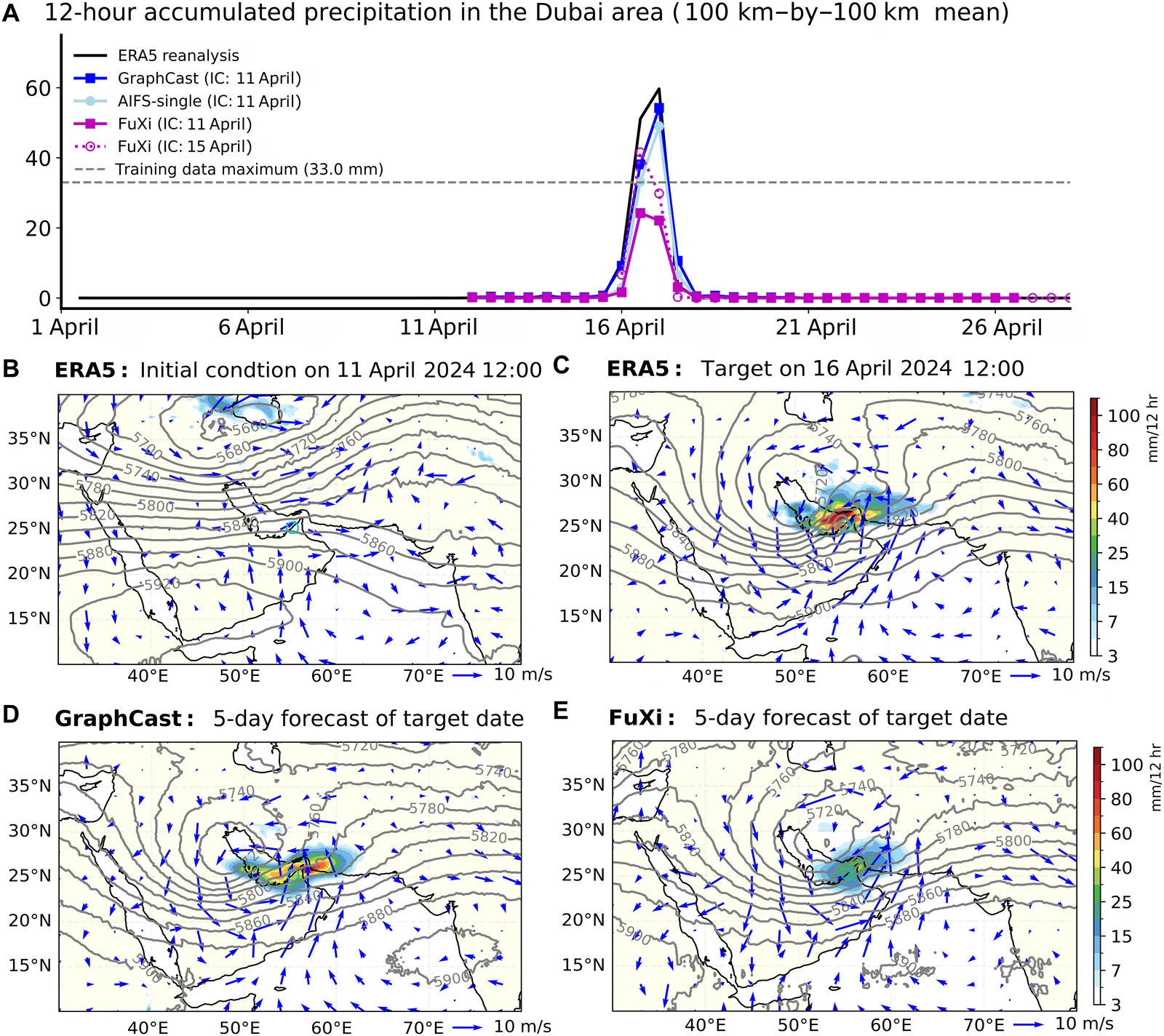
Forecasting Dubai’s 2024 extreme rainfall event using AI weather models. (**A**) Time series of 12–hour (hr) accumulated precipitation averaged around Dubai, comparing ERA5 reanalysis (black line) with forecasts from GraphCast (blue line; 5 days ahead), AIFS (light blue line; 5 days ahead), and FuXi (magenta line; 5 days ahead; dashed line, 1 day ahead). The horizontal dashed line indicates the maximum 12-hour accumulated precipitation (33 mm) in this region ever observed from 1979 to 2020, covering the training period for GraphCast, AIFS, and FuXi. (**B**) Circulation pattern and rainfall over the Arabian Peninsula on 11 April 2024, at 12:00 UTC (5 days before the event), showing 500-hPa geopotential height (contours), 850-hPa wind vectors (arrows), and 12-hour accumulated precipitation (shading), all from ERA5 reanalysis. (**C**) Same as (B), but for 5 days later, 16 April 2024 at 12:00 UTC, when the instantaneous rainfall value is close to its peak. (**D**) Same as (C), but for a 5-day forecast by GraphCast initialized on 11 April (B). (**E**) Same as (D), but for a 5-day forecast by FuXi. See fig. S2 for circulation forecasts from AIFS and fig. S3 for circulation forecasts from two other public AI models, Pangu and FourCastNetV2, which do not provide precipitation.

**Fig. 2.**
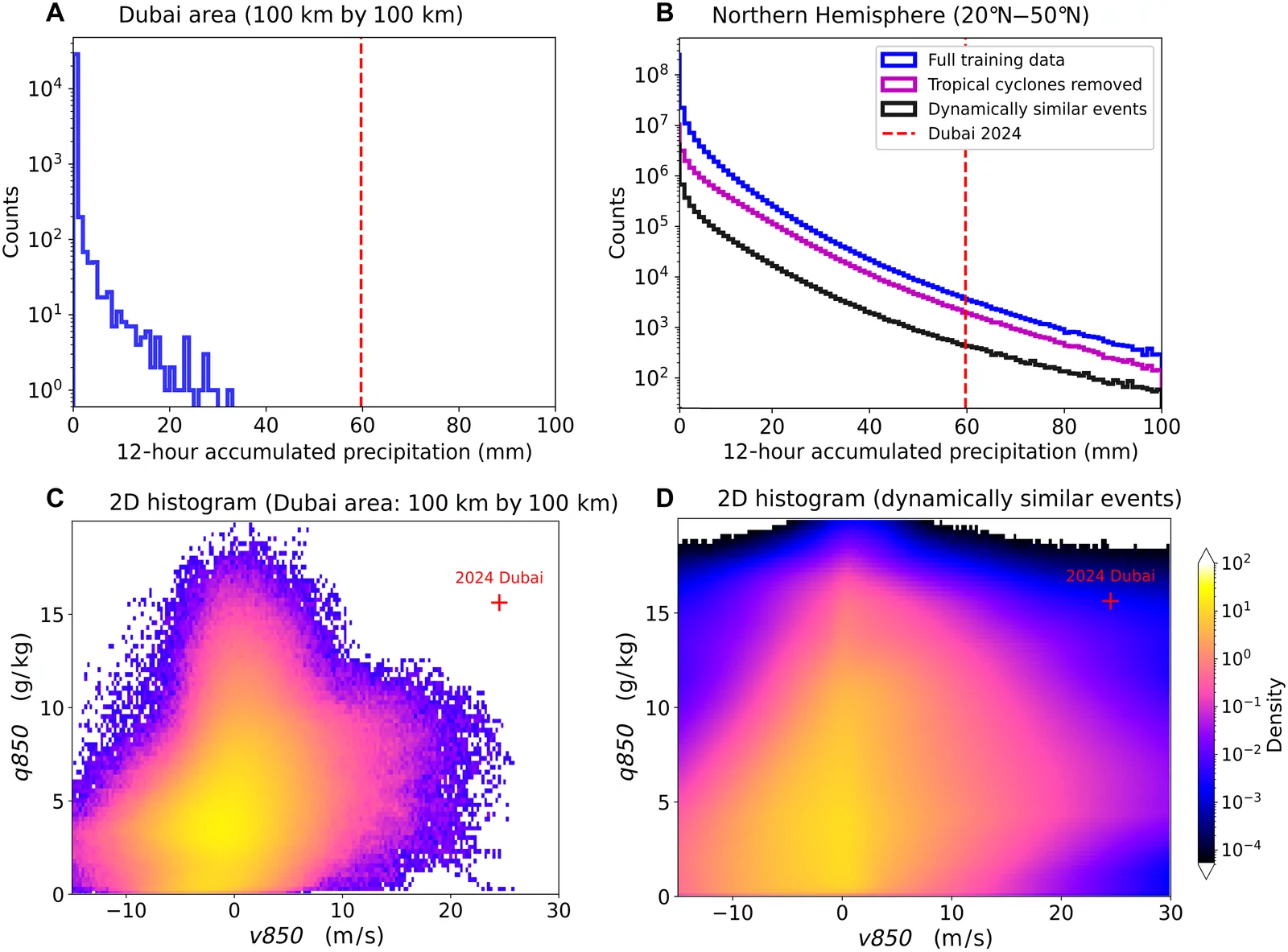
The Dubai rainfall event as an OOD event. (**A**) Histograms of 12-hour accumulated precipitation within a 100 km–by–100 km box around Dubai in the training dataset for GraphCast, AIFS, and FuXi models (ERA5, 1979–2020). (**B**) Same as (A), but focused on the 20°N to 50°N latitude band, considering the full training set and the same dataset but with tropical cyclones (TCs) excluded or only dynamically similar events (extratropical cyclones) included; see Materials and Methods for details. The vertical lines indicate the 12-hour accumulated precipitation during the 2024 Dubai extreme event. (**C**) Moisture transport around Dubai in the training set is shown as a 2D joint histogram of 850-hPa meridional wind (*v850*) and specific humidity (*q850*). The red “+” symbol marks the values of the 2024 Dubai event (15 to 16 April). (**D**) Same as (C), but for all dynamically similar events in the 20°N to 50°N latitude band.

While FuXi also captures the event’s timing, location, and circulation pattern (Fig. 1E), its 5-day forecast significantly underestimates the peak, producing values below the training maximum of 33 mm. Before further analyzing the performance of these AI models for this extreme event, below, we briefly discuss two issues: the fidelity of precipitation in ERA5 and the meteorological conditions through the evolution of this event. The latter discussion will help with understanding some of the later analyses in the paper.

The representation of precipitation in ERA5 is known to have shortcomings, mainly due to its coarse resolution and the lack of direct assimilation of relevant observations (*46*, *47*). However, for the purpose of this study, it suffices to examine the AI weather models within the “ERA5 statistical domain” since they are trained on ERA5 data (including precipitation), and we test them against ERA5 (including its precipitation) as the ground truth. Note that the shortcomings of ERA5 precipitation can be addressed in practice by training or fine-tuning the AI models on observation-derived datasets, such as IMERG (*48*).

To further understand the drivers behind the precipitation extremes, we examine the synoptic-scale meteorological evolution of the event. The textbook conceptual model of an extratropical cyclone is characterized by the convergence of warm and cold air masses and the associated precipitation near frontal boundaries (Fig. 3A). All elements of this conceptual model are present in the Dubai event (Fig. 3B). Briefly, the meteorological conditions 5 days before the event include a trough extending over the northern Arabian Peninsula, accompanied by southerly winds at 850 hPa, and light rainfall over Iran (Fig. 1B). During the peak of the event on 16 April, a fully developed cutoff low-pressure system is evident in the 500-hPa height field, centered over the Persian Gulf (Fig. 1C). The intensified meridional winds at 850 hPa facilitated substantial moisture advection into the region, resulting in concentrated areas of heavy rainfall, as indicated by 12-hour accumulated precipitation exceeding 100 mm in localized spots near Dubai. This value exceeded Dubai’s climatological annual accumulated rainfall, highlighting the severity of the event. Further analyses of the histograms of several key variables show that while the values of low-level meridional wind and specific humidity were at the tail of the marginal distribution (of the training set) around Dubai, they were not unprecedented (fig. S4); i.e., they were not OOD. What was unprecedented for this region was the northward moisture transport, which contributed to the OOD rainfall, as illustrated by the two-dimensional (2D) joint histogram of *v850* and *q850* (Fig. 2C). Note that *z500* (or its anomaly) and mean sea level pressure (mslp) were well in-distribution for this event (fig. S4).

**Fig. 3.**
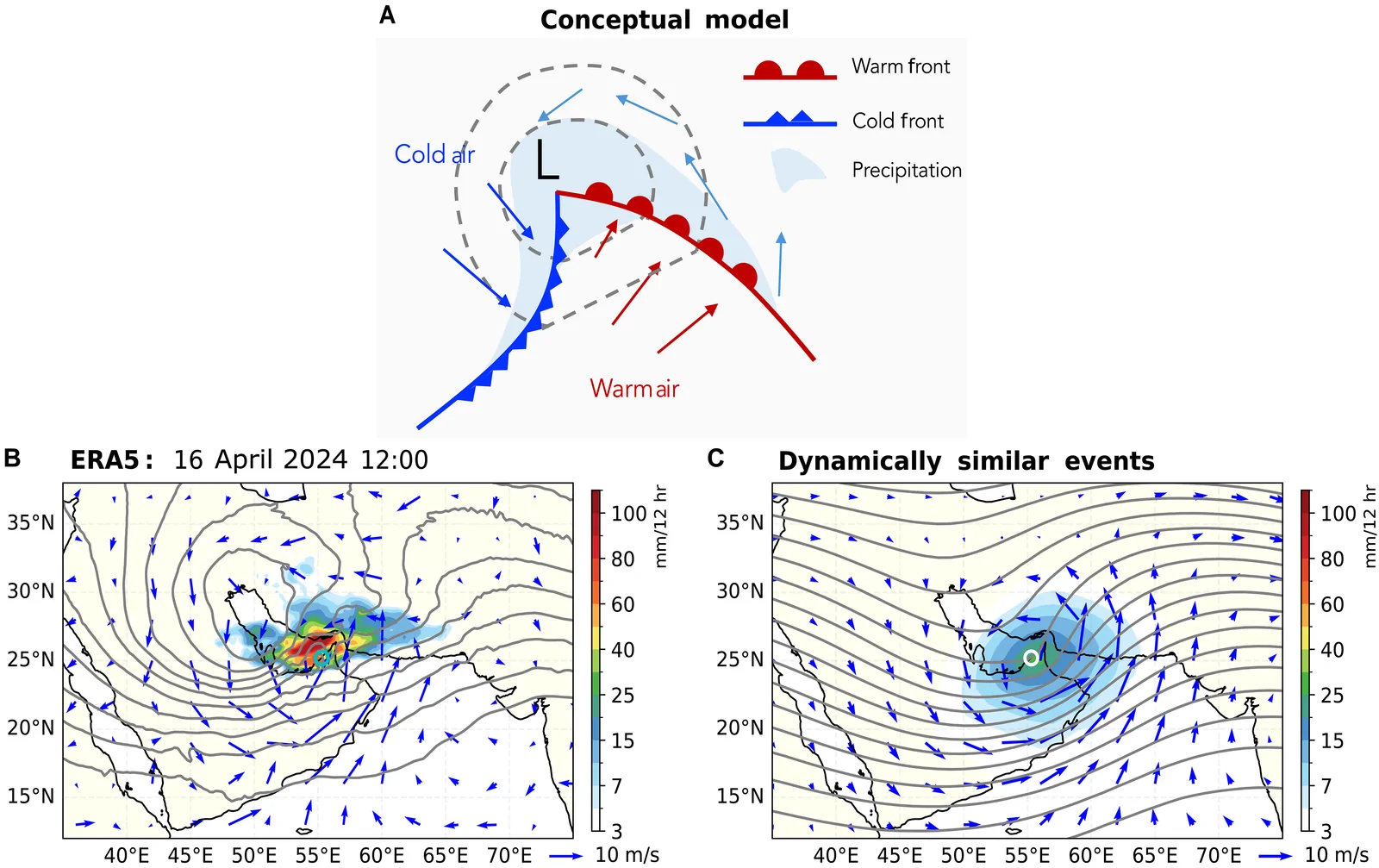
Dynamically similar events in other regions within the training dataset. (**A**) Conceptual model of an extratropical cyclone–driven heavy precipitation event, similar to the 2024 Dubai event. (**B**) Circulation and precipitation of the 2024 Dubai event in ERA5 (same as Fig. 1C). (**C**) Composite of dynamically similar events that have precipitation comparable to or even stronger than the Dubai event, identified from the training set in ERA5 over 20°N to 50°N latitude band of the Northern Hemisphere using a Lagrangian tracking algorithm (see Materials and Methods for more details). The patterns are centered on the city of Dubai for the composite.

It should be noted that the European Centre for Medium-Range Weather Forecasts (ECMWF) found their NWP model to also forecast this event quite accurately (*49*). As the focus of this paper is on the ability of AI weather models in forecasting OOD precipitation events, we did not analyze or present the NWP results.

### Factors contributing to the success of AI forecasts for Dubai’s unprecedented rainfall event

First, let us define a few key terms. As mentioned earlier, OOD in this study is defined as when the intensity of at least one input or output variable of the AI model exceeds any value seen in the training set in that region (grid point). We define OOD regionally to facilitate a clear definition of other terms below and because local information (e.g., latitude, longitude, and topography) is available to the AI models. This definition is based on the marginal distribution of each individual input or output variable. Therefore, the Dubai event was OOD because of the rainfall intensity (Fig. 2A). As Fig. 2C and fig. S4 show and as discussed in the broader AI literature (*34*, *50*), joint distributions (and measures such as the convex hull of the training data) can provide further information about the OOD behavior; however, these criteria are difficult to evaluate in practice for high-dimensional systems.

Furthermore, we distinguish two types of gray swans. A regional gray swan is OOD in a key variable such as surface temperature or rainfall, but dynamically similar events of comparable or greater intensity existed elsewhere in the world in the training data. A global gray swan is not only OOD but also more intense (in this key variable) than any dynamically similar events in the training set anywhere in the world.

For an AI model to successfully forecast an OOD event such as Dubai’s rainfall, there are two possible mechanisms:

1) Extrapolation—The model generalizes to intensity levels that exceed the local training maximum, using information from weaker, local events. As an example, in this mechanism, the model correctly forecasts Dubai’s unprecedented rainfall based on weaker, more frequent local similar events that it has seen near Dubai in the training set.

2) Translocation—The model leverages dynamical similarities across different geographic regions. In this mechanism, the model learns to predict Dubai rainfall from comparable or stronger dynamically similar events occurring in other regions (spatially nonlocal) in the training set.

Although both mechanisms involve a form of OOD generalization, we distinguish them to decouple the challenge of predicting unprecedented intensities at a given location using weaker, local events versus using stronger, nonlocal events in the training set. Extrapolation is the commonly hypothesized mechanism for the potential success of AI weather models with gray swans. Translocation is a mechanism that emerged from a recent study by some of the current authors and their collaborators (*25*). That study, which was inspired by this analysis of the Dubai unprecedented rainfall event, trained versions of another AI weather model (FourCastNet) with modified training sets. It was shown that the AI model cannot forecast category 5 tropical cyclones (TCs) if it has not seen any category 3 to 5 TCs in its training set, a result that demonstrates a lack of extrapolation. However, it was also shown that the version trained with category 3 to 5 TCs excluded from only one ocean basin (Atlantic or Pacific) has skill forecasting category 5 TCs everywhere, including in that basin, suggesting that the AI model can generalize across tropical basins (i.e., translocate) despite the presence of location encoding. They further argued that translocation only works among events with similar dynamics, which is manifested to the AI model through the relationship between variables in the entire state vector.

Below, we will first present an in-depth analysis of learning from translocation, followed by an investigation of extrapolation. Note that these two mechanisms are not mutually exclusive.

#### Learning via translocation of stronger dynamically similar events

As shown in Fig. 2 (A and C), this event was a regional gray swan in terms of the training dataset’s distribution of 12-hour accumulated precipitation around Dubai, resulting from an unprecedented northward moisture transport. However, across the broader Northern Hemisphere, precipitation events of similar or higher magnitude exist in the training set (Fig. 2B). Recognizing that some of the highest precipitation values are associated with TCs moving into the extratropics, we conduct additional analyses to exclude TC-induced precipitation (pink line in Fig. 2B). Furthermore, we isolate precipitation only from events that have a meteorological driver similar to those of the Dubai event, that is, an extratropical cyclone. Details of this analysis can be found in Materials and Methods. Briefly, using a Lagrangian tracking algorithm, we identify dynamically similar events with precipitation that is comparable to or higher than the Dubai event in the training set. The circulation and precipitation composites of these events closely resemble those of the Dubai event (Fig. 3C), albeit with reduced amplitude due to the smoothing effect of averaging many cases.

Figure 2B shows that many events with dynamics similar to that of the Dubai event but with comparable or higher precipitation existed in other regions of the training set of the AI models. The same is true for moisture transport, as demonstrated in the 2D joint histogram of *v850* and *q850* (Fig. 2D). This shows that the key dynamical process of this event was “in-distribution” when the entire Northern Hemisphere is considered.

These results suggest that while the 2024 event was unprecedented for the Dubai area (OOD), many dynamically similar events existed elsewhere in the Northern Hemisphere within the training set, potentially providing learning opportunities for such a “regional” gray swan via translocation. Building on the findings of Sun *et al.* (*25*), we speculate that this is at least one way for GraphCast and AIFS to skillfully forecast the Dubai event. Note that demonstrating translocation unambiguously following the framework of (*25*) requires training the AI model from random weights with a modified dataset (e.g., with all samples containing stronger dynamically similar events removed from other regions). However, this procedure is nontrivial and computationally very expensive for GraphCast or AIFS and is not pursued here. Instead, in this work, we first leverage a diagnostic tool based on deep learning theory to understand if GraphCast and AIFS could use spatially nonlocal information as they learned about the Dubai area’s precipitation. Then, we introduce a causal experiment based on fine-tuning on a modified training set to further test the translocation mechanism.

For a convolutional neural network, the “receptive field” is the region of the input that the network can use/see to predict the output (*51*, *52*). Theoretically, the receptive field can be derived from the network depth, convolutional kernel size, and other architectural choices. It quantifies how much spatially nonlocal information from the input the network can, in principle, use to produce an output. For any architecture, this can be numerically estimated using the ERF [see Pahlavan *et al.* (*52*) for an example in climate modeling]. More specifically, ERF is computed by backpropagating a signal from the output layer to the input layer. ERF quantifies the influence of different input regions on a given output (specific variable at a specific location)—similar to what adjoint methods do, which, for example, have been recently used to estimate forecast sensitivity in AI models to initial conditions (*33*, *53*). See Materials and Methods for details of the ERF computation.

The ERFs of GraphCast and AIFS, both of which demonstrate strong performance for this event, for a one-time-step (6-hourly) forecast of precipitation around Dubai are shown in Fig. 4 for selected variables that play a role in the dynamics of extratropical cyclone-driven precipitation. The analysis shows that while GraphCast mainly relies on local information, as expected, it also exhibits a nonlocal, in fact “global”, ERF that aligns with the multi-icosahedral mesh of GraphCast. For AIFS, its ERF exhibits a pronounced nonlocal latitude band pattern, reflecting the model’s attention window design (*5*). Both ERFs show substantial nonlocality that, in almost all analyzed variables (e.g., *v850*, *q850*, and *z500*), covers a large area from the east Atlantic to the east Pacific. We note that this is the almost instantaneous ERF (from a one–time step, 6-hourly forecast); it is not a result of the advection and propagation of waves and teleconnection to the Dubai area over time. The highly nonlocal ERFs suggest that the model architecture has the capacity to draw on information from distant regions for local precipitation predictions. Note that a nonlocal ERF does not necessarily mean nonlocal learning. Whether this architectural capacity is actually exploited during training is tested directly by the fine-tuning experiment described below.

**Fig. 4.**
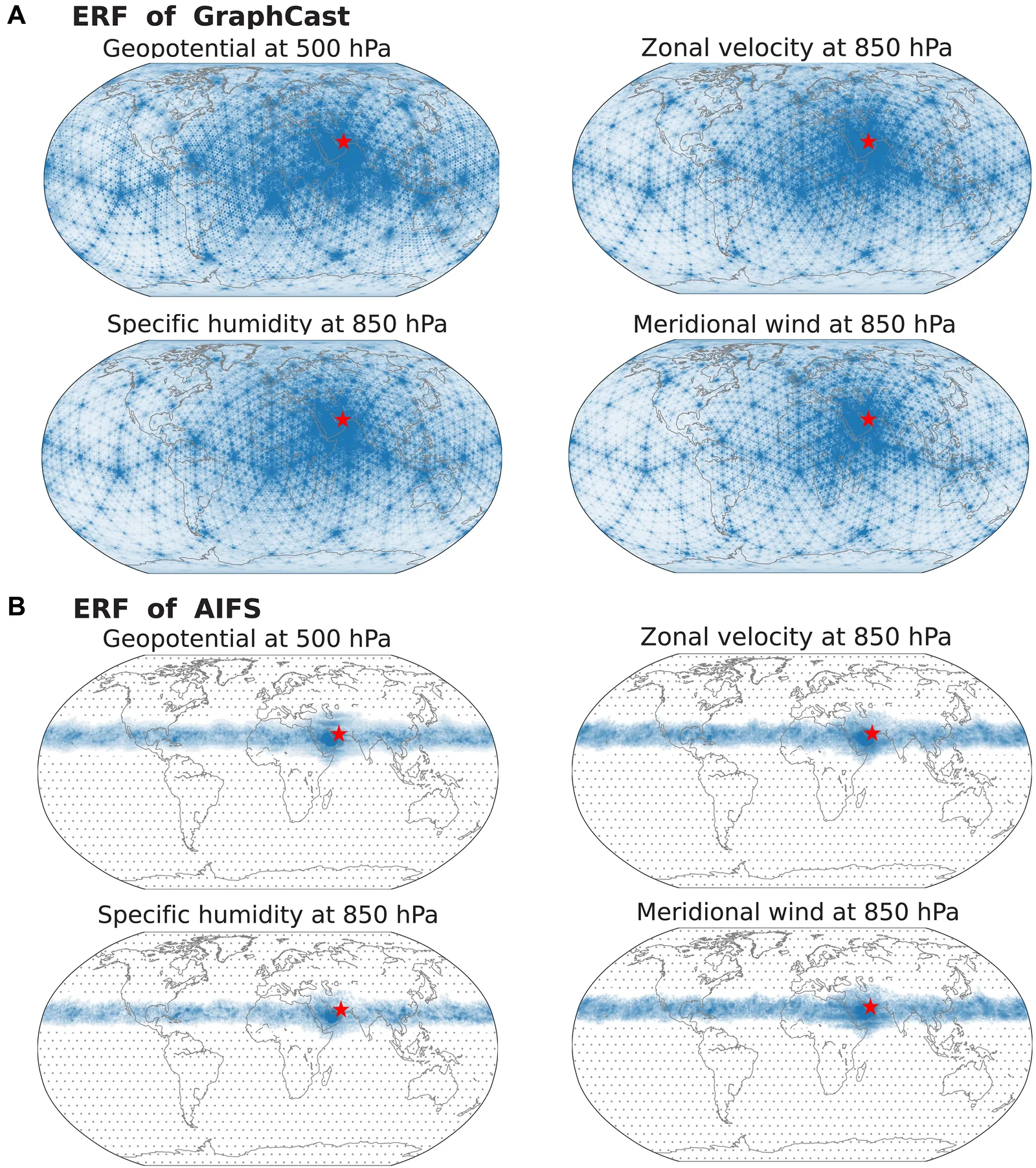
ERF of GraphCast and AIFS for forecasting precipitation (6 hours later) in the Dubai area. Four selected variables are shown: geopotential at 500 hPa, zonal velocity at 850 hPa, *q850*, and v850. The forecast is initialized at 12:00 UTC on 15 April 2024, with ERF calculated after one time step (6 hours). The star symbol in each panel shows the location of Dubai. GraphCast uses two previous time steps for predictions; we show results for the later time step, as the earlier step exhibits a similar but weaker pattern. Dot stippling in (B) indicates regions where the field is zero (absolutely no information is used for the prediction). The maps are scatter plots where point size and color intensity both scale with the standardized ERF amplitude, with darker blue regions indicating stronger influence on precipitation predictions over Dubai. Different from the actual gradient calculation, only relative spatial variations of the ERFs are meaningful, so we omit a color bar and physical units. The multi-icosahedral mesh structure is distinctly visible in GraphCast, while AIFS results show a latitude band, consistent with the model’s attention window design (*5*). See Materials and Methods for details of ERF calculations.

To further test the translocation hypothesis without the prohibitive cost of training the models from scratch [which was used in (*25*)], we develop an efficient, causal experiment based on fine-tuning, leveraging the machinery for GraphCast provided by Subich (*54*). Here, we design a sensitivity experiment in which we modify the training dataset by capping precipitation at 30 mm per 12-hour period for all dynamically similar events outside the Dubai area (this value is motivated by the historical maximum in the Dubai region in the training set; see Fig. 1A). GraphCast is then fine-tuned on this modified dataset (see Materials and Methods for details). The idea behind this experiment is as follows: If the model predicts the extreme 2024 Dubai event via extrapolation (at a fixed location), then capping precipitation values outside Dubai should not affect the forecast skill, as the local training data for Dubai remain unchanged. In contrast, if the model relies on translocation, then suppressing the intensity of nonlocal analogs should degrade the forecast skill for the 2024 Dubai event.

Our results confirm the translocation mechanism (Fig. 5). When dynamically similar events in other regions are restricted to Dubai’s historical maximum, the fine-tuned model’s forecast for the 2024 Dubai event drops to slightly above 30 mm, significantly underestimating the observed intensity and the original forecast (Fig. 5A). This failure is not due to a loss of skill for dynamics: Figure 5B confirms that the model still correctly predicts the large-scale circulation pattern associated with the event (comparable to the original successful forecast in Fig. 1D). The global accuracy metrics also show minimal change across 72 test cases in 2024, indicating consistently strong performance for other variables after the fine-tuning (Fig. 5). The rainfall forecast distribution in the tropics, where the data were not modified and the dynamics of precipitation extremes are different, remains largely unaffected (fig. S5). This also confirms that the degradation is specific to the target extreme event and not a result of general model deterioration.

**Fig. 5.**
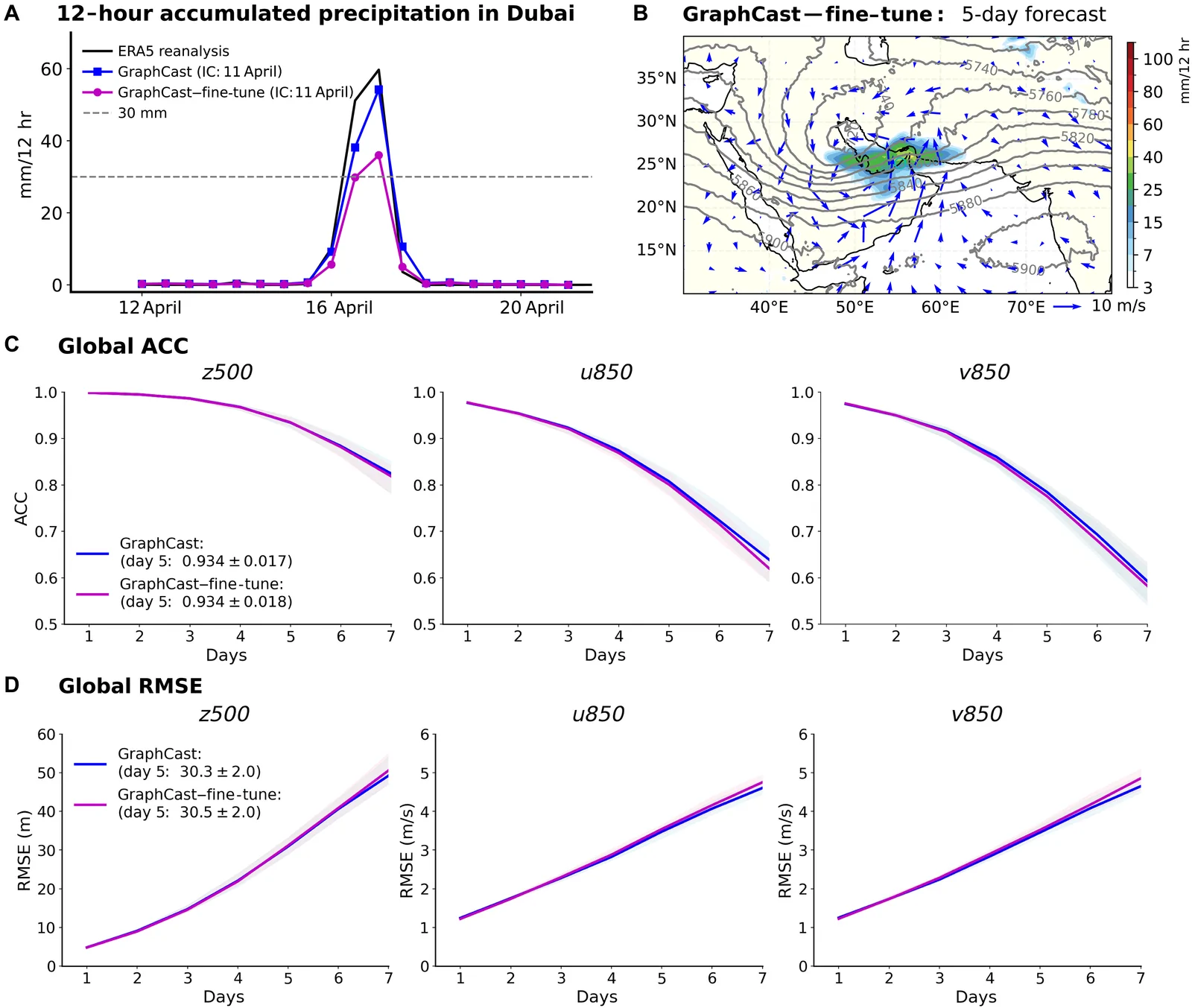
Fine-tuning GraphCast with samples that have the precipitation of dynamically similar events outside Dubai, capped degrades the forecast skill for the 2024 Dubai event. (**A**) Forecast of the 2024 Dubai rainfall event by the original GraphCast model versus the version fine-tuned on data where precipitation for dynamically similar events outside the Dubai region was capped at 30 mm per 12-hour period (approximating Dubai’s historical maximum). (**B**) Circulation pattern of the Dubai event predicted by the fine-tuned model (analogous to Fig. 1D), showing that the circulation forecast skill remains intact despite the decline for rainfall forecast skill. (**C**) Global anomaly correlation coefficient (ACC), and (**D**) Global root mean square error (RMSE) for the original and fine-tuned models. Global metrics are averaged over 72 test cases sampled every 5 days throughout 2024, demonstrating that the fine-tuning process did not degrade general global forecast skill.

Although we do not conduct similar fine-tuning experiments with AIFS due to technical constraints, the results from GraphCast strongly suggest that the model’s skill in forecasting regional gray swan extremes depends critically on its ability to draw on intensity information from geographically distant but dynamically similar events (i.e., to translocate). The nonlocal ERFs identified above provide one architectural pathway through which information from remote regions could be leveraged for predictions over Dubai. This pathway could operate alongside other mechanisms that support nonlocal learning, including spatial weight sharing intrinsic to neural network architectures (*3*, *55*) and the capacity to represent physical processes that are only weakly region dependent (*33*, *56*). Together, these mechanisms may contribute to the transfer of information from stronger, dynamically similar events and help explain skillful prediction of regional gray swans.

#### Evidence against learning via extrapolation from weaker local events

The earlier analysis suggests that the AI models might have learned to forecast the Dubai event, a regional gray swan, via translocation. However, this does not exclude the possibility of extrapolation from weaker events.

To further evaluate this possibility of extrapolating beyond the local training maximum, we analyze GraphCast’s performance on dynamically similar events from across the Northern Hemisphere (20°N to 50°N) with 12-hour accumulated precipitation higher than Dubai’s. The highest precipitation from an extratropical cyclone in the ERA5-based training set was 126 mm in 12 hours. We select the top five 12-hour accumulated precipitation events from the testing set (2020–2023), which have maximum precipitation around 90 to 100 mm in 12 hours. Thus, none are global gray swans, but they are in-distribution at the very end of the tail (see Fig. 2B). One such event occurred in Florida in December 2023 (Fig. 6A). This event, associated with a cyclone accompanied by frontal winds, is dynamically similar to the Dubai event. However, GraphCast’s 5-day forecast significantly underestimates the rainfall, with peak values close to 60 mm (Fig. 6B). This underestimation of accumulated precipitation extremes is consistent across all five examined extreme events (Fig. 6C and fig. S6). While GraphCast has already seen even stronger dynamically similar events in the training set (with around 126 mm per 12-hour precipitation rate), its 5-day forecasts have maxima between 30 and 60 mm per 12-hour rate, much lower than the ∼90 mm per 12-hour rate of the actual events.

**Fig. 6.**
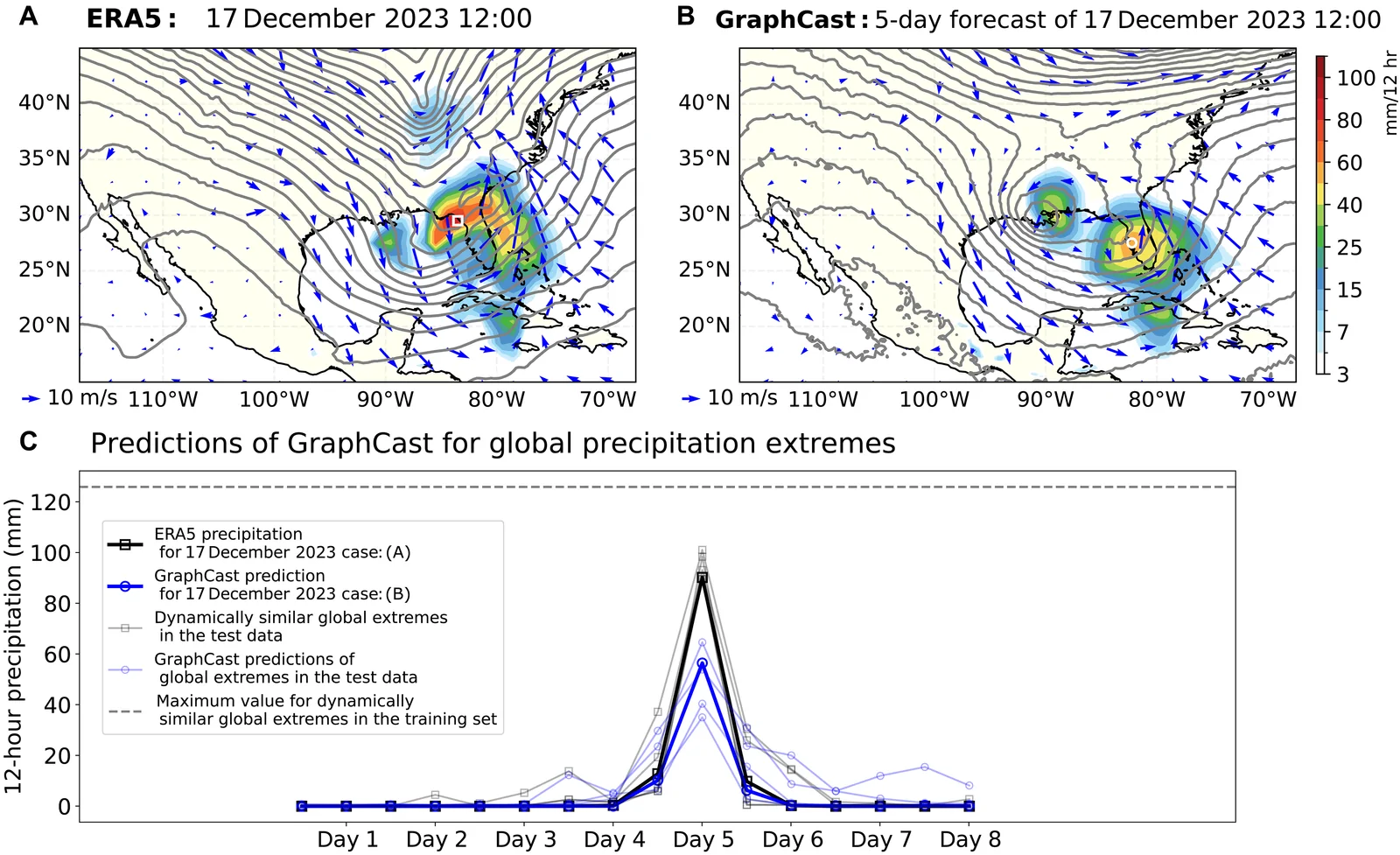
GraphCast’s limitations in accurately predicting highest amplitude precipitation extremes of the Northern Hemisphere. (**A**) similar to Fig. 1A, but for one of the top five extratropical cyclone–driven extreme precipitation events of the test set (2020–2023). This event happened in December 2023 over Florida, USA, with 12-hour accumulated precipitation reaching 90 mm. (**B**) Same as (A), but for a 5-day forecast with GraphCast. (**C**) Time series of 12-hour accumulated precipitation for the top five dynamically similar events (see fig. S6 for the circulation and precipitation patterns of the other four cases). These events all have 12-hour accumulated precipitation exceeding 90 mm. The black and gray lines represent the truth in the ERA5 reanalysis, while the blue (light blue) lines represent the 5-day forecasts of GraphCast. The white square and circle denote the locations of maximum precipitation in the ERA5 reanalysis and GraphCast, respectively. In (C), the time axis has been shifted such that day 5 aligns with the day of maximum precipitation in ERA5 for each of the five events. The maximum precipitation for all the dynamically similar events in the training data (1979–2017) is marked with the gray dashed line.

The inability of GraphCast to reproduce the tail of the extratropical precipitation distribution can be seen in the histograms of Fig. 7. This shortcoming is even more pronounced in the tropics, where the precipitation amplitudes are higher. The same figure shows that the difficulty with reproducing the tail of the extratropical and tropical precipitation distribution is much more severe for FuXi. The problem worsens for both models at longer lead times (Fig. 7); see the “Spectral bias as a limiting factor for the skill of AI models for extreme precipitation forecasting” section for further analysis and discussion. Note that AIFS was released publicly only recently and uses a graph neural network architecture with performance comparable to GraphCast. Therefore, our analysis of AIFS is limited to essential diagnostics related to translocation, while a more comprehensive investigation (e.g., of extrapolation) is left for future work.

**Fig. 7.**
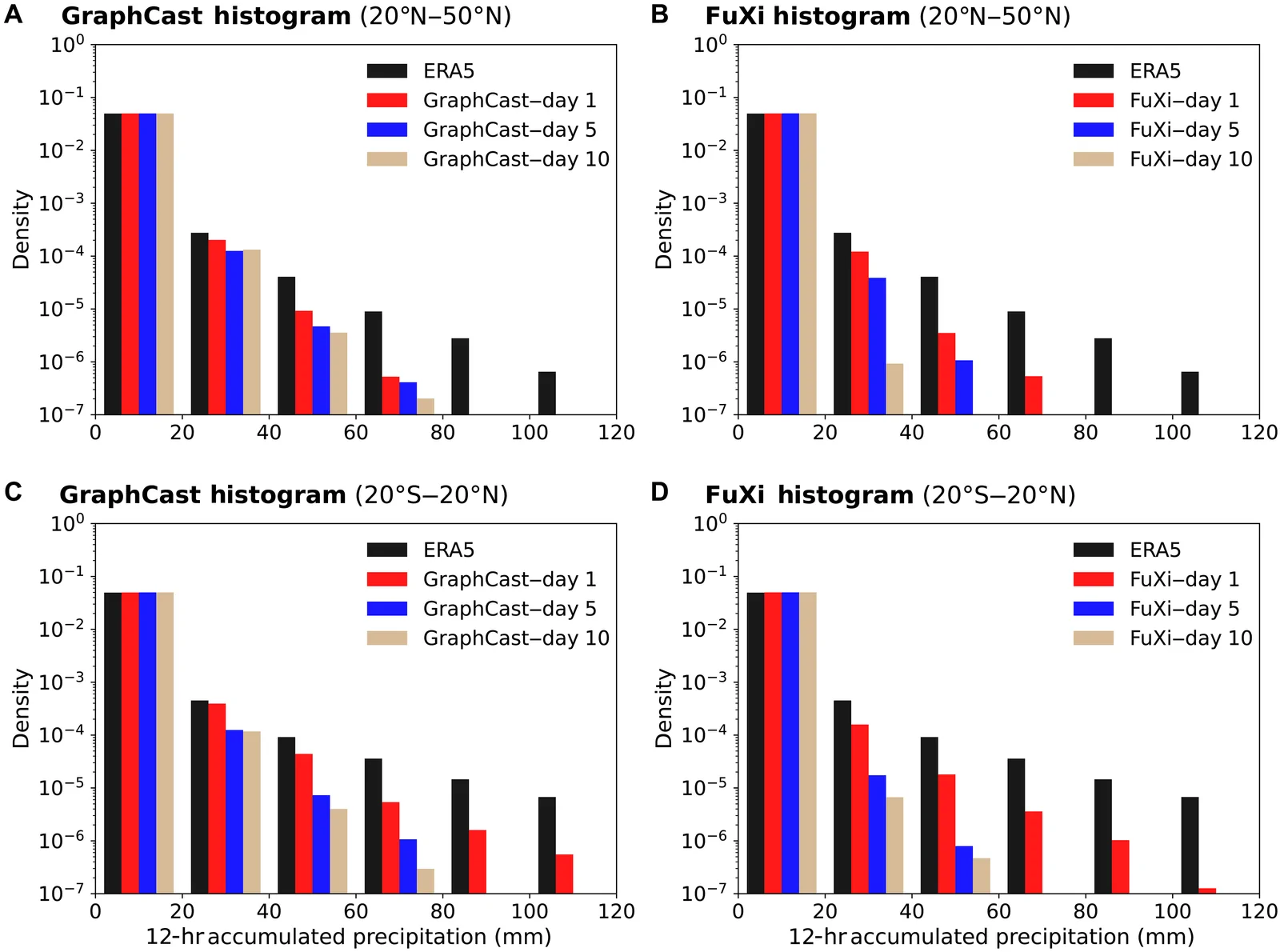
Poor representation of 12-hour accumulated precipitation distribution’s tails in the 1-, 5-, and 10-day forecasts of GraphCast and FuXi. Histograms represent aggregated forecasts initialized daily throughout April 2024. (**A** and **B**) GraphCast and FuXi in the 20°N to 50°N latitude band of Northern Hemisphere. (**C** and **D**) GraphCast and FuXi in the 20°S –20°N tropical latitude band. Note that these histograms include all events (not just dynamically similar events).

So far, the results show that GraphCast severely underestimates the strongest global extreme precipitation events, which, although rare, still fall within the distribution of the training data. FuXi exhibits a similar tendency to underestimate the precipitation distribution tail, as shown in the histogram analysis (Fig. 7). Together with the fine-tuning result, which shows that GraphCast fails to reproduce the Dubai event once rainfall in dynamically similar events is capped, these findings suggest that extrapolation from weaker events is not a viable mechanism, as these models struggle even with infrequent yet in-distribution events. This might appear as strong evidence for a data imbalance problem. However, as briefly discussed next, at least some of this underestimation might be due to smoothing from spectral bias.

### Spectral bias as a limiting factor for the skill of AI models for extreme precipitation forecasting

To better understand the difference in the performance of GraphCast and FuXi, we next examine the spectral bias of each model. As discussed in Introduction, spectral bias (*39*, *57*) is a common challenge for AI models, including the recent AI weather models (*38*, *58*–*60*): They preferentially learn large-scale features while struggling with the small-scale ones. Before discussing the root cause of this problem and its implications, we first show the representation of small scales (high wave numbers) in the forecasts of GraphCast and FuXi, especially for precipitation. Figure 8 (A to C) presents the spectral power of 12-hour accumulated precipitation at 1-, 5-, and 10-day forecast lead times. At a 1-day lead time, spectra of forecasts from both GraphCast and FuXi show reasonable alignment with ERA5’s at lower wave numbers, which correspond to large-scale features, although with slightly lower spectral power (worse for FuXi). However, at higher wave numbers (smaller scales), both models exhibit a noticeable underestimation of spectral power. This underestimation indicates that even for short forecasts, both models struggle to capture finer-scale precipitation details.

**Fig. 8.**
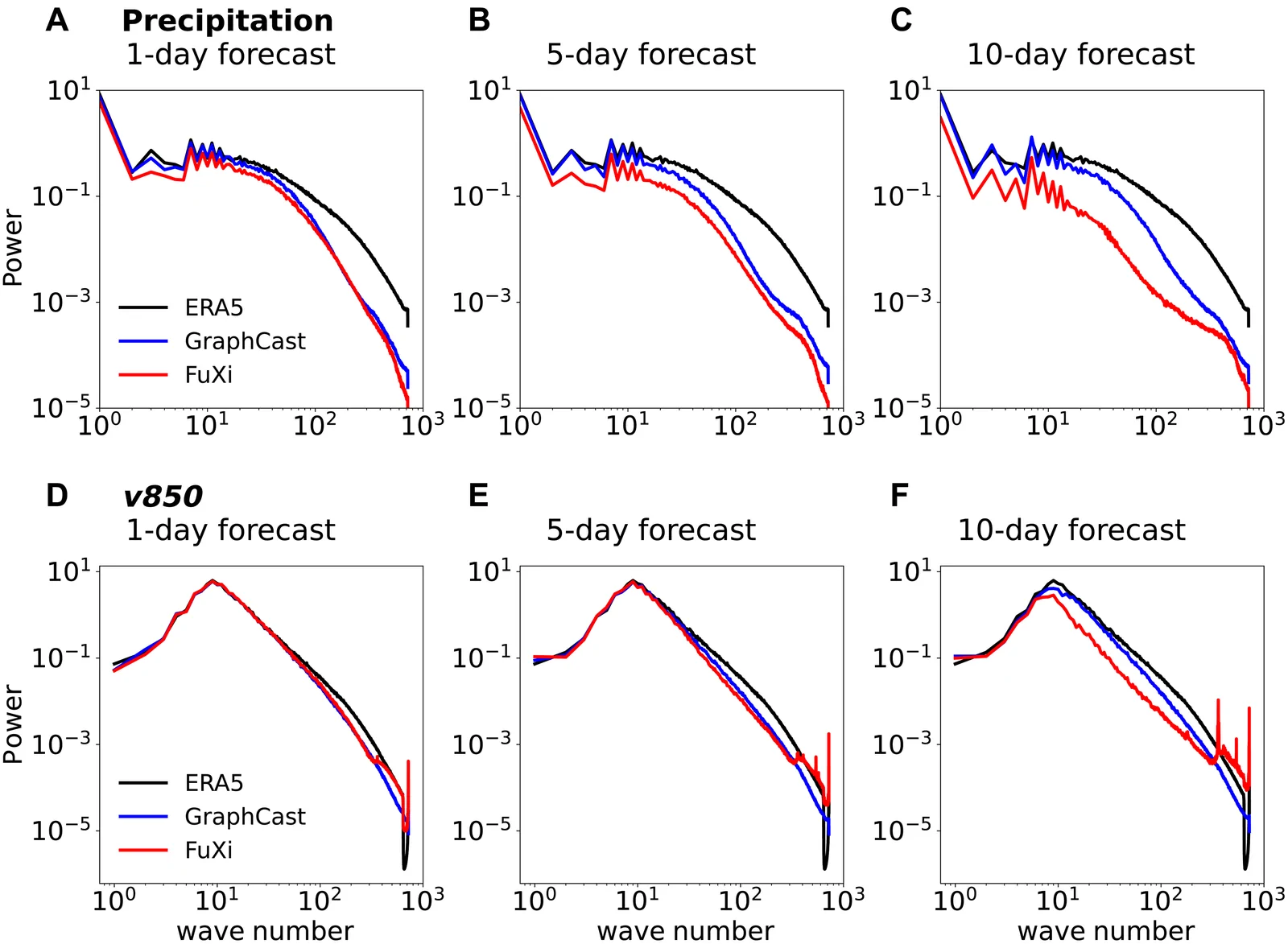
Poor representation of small scales in GraphCast and FuXi forecasts. (**A** to **C**) Spectral power of 12-hour accumulated precipitation and (**D** to **F**) v850 (*v850*) as a function of wave number. The ground truth (ERA5 reanalysis) and forecasts from GraphCast and FuXi for different forecast lead times are compared. Spectral analysis using spherical harmonics is conducted for daily initialized predictions of global fields in April 2024; hence, the results shown here are the average of 30 spectra.

At longer lead times of 5 and 10 days (Fig. 8, B and C), the underestimation of spectral power in GraphCast’s forecasts only slightly worsens. However, FuXi’s spectra further deteriorate as the lead time increases, resulting in a significant underestimation of the power at small scales and even large scales on day 10 (Fig. 8C). The loss of spectral power across the scales can lead to the poor representation of extreme events. This analysis explains FuXi’s diminishing ability to capture the tail of the precipitation distribution as the lead time increases (Fig. 7). To see the implication for Dubai’s rainfall event, we show the time series for the 1-day forecast with FuXi in Fig. 1A. Unlike the 5-day forecast, which only reached around 25 mm, the 1-day forecast is close to GraphCast’s 5-day forecast in terms of onset and amplitude, peaking at 44 mm, above the 33 mm limit in the local training data. This suggests that the significantly underestimated peak in FuXi’s 5-day forecast is largely attributable to the smoothing caused by spectral bias rather than to the event’s rarity (data imbalance). Note that spectral bias is also evident in the spectra of other variables, such as v850 (Fig. 8, D to F), but it is most pronounced for precipitation, which is dominated by small-scale features. This exacerbates the challenge of forecasting the inherently multiscale phenomena where small-scale processes play an important role.

The above analyses show that spectral bias is a major limiting factor in forecasting extreme precipitation events, especially at longer lead times. Before further discussing why the magnitude of this bias can vary substantially between AI models, it is essential to explain its root causes.

Spectral bias, also known as the frequency principle, is a well-known problem in deep learning theory rooted in the optimization process during training (*39*, *57*). Spectral bias is ubiquitous in applications of neural networks to multiscale patterns, ranging from reconstruction of static images, e.g., of animals (*61*, *62*), to prediction of climate data (*58*, *60*, *63*, *64*). Chattopadhyay and Hassanzadeh (*58*) discuss the theoretical and practical aspects of spectral bias in climate and weather applications in detail. Although spectral bias is usually seen as only a loss function problem [especially of mean squared error (MSE)–type losses], theoretical work shows that spectral bias strongly depends on the activation function as well (*57*).

Specific to weather and climate prediction, two additional factors contribute to spectral bias. First, in a chaotic atmosphere, small scales are less predictable than large scales. This issue, in particular, affects AI models that are trained with multiday rollouts, i.e., using several consecutive forecast steps rather than just a single step in the loss function during training, as done in GraphCast (up to 3 days) and FuXi (up to 13 days for FuXi-Long given the cascade learning used there). If the rollout horizon exceeds the predictability limit, the model might attempt to suppress small-scale variability altogether, further reinforcing spectral bias. Second, when small-scale predictions do not precisely align with the observed spatial location, they incur a disproportionately high penalty—a phenomenon known as the “double-penalty” effect (*38*, *65*). This discourages models from attempting to predict small-scale variations, leading to smoothing.

The more severe spectral bias in FuXi likely stems from the model’s neural network architecture and optimization strategy, and, in particular, the special adjustments to reduce MSE-type loss over longer lead times, which, as discussed above, can suppress the small scales. Overall, spectral bias can be a major hindrance to accurately forecasting the peak amplitude of extreme precipitation events. Several general remedies for spectral bias in weather and climate models have emerged in recent years, e.g., by regularizing loss functions to better learn small scales, using multistage training, and using diffusion models (*6*, *7*, *38*, *58*, *63*, *64*).

## DISCUSSION

The key findings of the paper are as follows:

1) GraphCast and AIFS are able to forecast the location, timing, and the amplitude of the unprecedented 2024 Dubai rainfall and the associated circulation pattern remarkably well, even at an 8-day lead time (Fig. 1 and figs. S1 and S2). FuXi demonstrated similar skill, although it tends to significantly underestimate the rainfall amplitude except at much shorter lead times.

2) The success of the AI models in this event is particularly notable, given that the event’s rainfall intensity was substantially out of the training distribution for that region (Fig. 2).

3) GraphCast’s success stems from translocation, the ability of the model to learn from dynamically similar events in other regions during training. Fine-tuning GraphCast on modified training data for which values of precipitation of dynamically similar events are capped outside Dubai (but unchanged around Dubai) substantially reduces the precipitation forecast skill of the 2024 Dubai event, providing causal evidence that the model relies on translocating from remote analogs rather than local extrapolation (Fig. 5). The neural network’s nonlocal ERF (Fig. 4) indicates the architectural capacity to draw on information from distant regions.

4) The limited ability to extrapolate from weaker events, data imbalance, and spectral bias are major limiting factors for the AI models in dealing with globally rare or unprecedented weather extremes (Figs. 6 to 8).

Below, we further discuss findings (3) and (4) and their practical and theoretical implications for AI weather models and the emerging AI emulators of long-term atmospheric and oceanic variability built on them (*66*–*68*).

### Success via translocation and its implications

We show that GraphCast and AIFS have nonlocal ERFs, indicating that the model architecture has the capacity to leverage information from remote regions (Fig. 4). At the same time, we demonstrate that while the Dubai event’s rainfall intensity was significantly OOD with respect to the local (Dubai area) conditional training distribution, it was within-distribution when considering globally similar dynamics (Fig. 2), illustrating the regional versus global gray swan contrast that is central to this study. Capping precipitation in these dynamically similar events directly affects GraphCast’s performance in Dubai, as demonstrated in the fine-tuning experiments. Taking all the results together and combining the insight from the controlled experiments of Sun *et al.* (*25*), we infer that the success of GraphCast and AIFS in forecasting this regionally unprecedented (OOD) event results from translocation. Note that we did not compute the ERF of FuXi due to computational constraints; while the ERF of deep, large neural networks such as the one used in FuXi is expected to be nonlocal (*52*), this speculation has to be further tested in future work.

The ability of these AI models to learn from events in other regions is encouraging from a practical perspective. It suggests that the models may be capable of short-term forecasting and long-term emulation of regionally unprecedented events that arise from natural variability or shifting weather patterns due to climate change, provided that similar events have occurred elsewhere in the training data. Moreover, this transferability may offer significant value for regions with limited historical observations, such as Africa where precipitation records are sparse (*69*). Nevertheless, more work is needed to systematically evaluate the performance of AI weather and climate models on these fronts. Diagnostic tools, such as ERFs, and controlled experiments, such as the fine-tuning tests in this work and experiments introduced in previous studies (*25*, *56*, *70*), offer promising pathways for advancing this understanding.

We also note that a nonlocal ERF alone does not guarantee translocation. The translocation ability of the neural network might stem from other factors too, such as shared weights in GraphCast across different regions (*3*, *55*) and the AI models’ capacity to capture the underlying physical processes that might have less dependency on the regions (*33*, *56*). Note that these mechanisms are not mutually exclusive. Methods such as adjoint-based analyses analogous to the ERF (*71*) and studies of neural networks’ weight evolution during training (*72*) may also provide valuable insights into the mechanisms underlying the generalization capacity and translocation ability of neural networks.

From a theoretical perspective, the ability to translocate suggests that AI models learn generalizable physical relationships and statistical structures, rather than overfitting to location-specific data. This finding raises an important question about these AI models’ internal representation. From the perspective of the state space distribution conditioned on location, the 2024 Dubai event was OOD, successfully predicted via translocation. However, from the perspective of the models’ learned latent representation, the picture may not be so clear. If GraphCast encodes geographic location in a spatially transferable manner, for instance, via positional encodings or the shared weights of its graph network architecture, then dynamically similar events from distant regions may map to nearby points in the model’s latent space. Under this view, “translocation” in state space corresponds to “interpolation” in latent space. While this is currently only a speculation, further probing the models’ internal representations will be an interesting direction to pursue in future work. Nevertheless, this framing is consistent with the growing evidence that AI weather models learn physically meaningful spatiotemporal relationships (*33*, *56*, *70*, *73*).

An interesting question is how translocation can be further exploited in the design of the next generation of AI weather and climate models. Recently, ERFs have been discussed in a few studies on weather/climate modeling. Most notably, Karlbauer *et al.* (*74*) estimated the physically meaningful size of receptive fields in AI models of the atmosphere based on the speed of sound (∼300 m/s) and maximum speed of the jet stream winds (∼100 m/s), which, for a 6-hourly time step, yield around 6500 and 2150 km in each direction, respectively. Along the same lines of connecting the ERF size with atmospheric dynamics, Pahlavan *et al.* (*52*) examined various emulators of a 1D model of quasi-biennial oscillation, concluding that extensively nonlocal ERFs are needed to capture nearly instantaneous long-range correlations that can arise from the interaction of the mean flow with fast propagating processes such as gravity waves. The results of the current study, however, suggest a potential benefit in having large ERFs, beyond what would be physically meaningful spatial scales, to help translocation. Nonetheless, large ERFs can also increase the likelihood of learning spurious and nonphysical relationships, warranting further investigation into the best choices for ERFs.

### Limiting factors and the need for innovations

The three aforementioned limiting factors (spectral bias, data imbalance, and lack of extrapolation) have different drivers and likely require different innovative solutions. Addressing spectral bias has received considerable attention in recent years (*6*, *38*, *58*, *63*, *64*, *75*, *76*). In particular, the use of diffusion models, e.g., in GenCast (*7*), appears to significantly reduce the spectral bias. Future work should further investigate how these approaches improve the representation of the dynamics (rather than just statistics) of the small-scale processes.

Data imbalance for in-distribution events can potentially be mitigated by weighting the loss function (*77*–*80*). For globally OOD events, a promising approach is oversampling, i.e., generating stronger extreme events using physics-based models guided by techniques such as rare event sampling (RES) algorithms (*81*–*84*) and ensemble boosting (*85*) for the retraining of the AI model. Note that AI models themselves can accelerate RES algorithms (*23*), thus creating a feedback loop between the RES algorithm, the AI model, and the physics-based model.

Last, the question of extrapolation from weaker events requires extensive and careful investigations. In particular, one has to disentangle extrapolation and translocation, as, for example, in the case of the Dubai event, what might appear as extrapolation is in fact translocation. Thus, evidence of extrapolation should focus on events that are at the tail of, or out of, the global distribution of the training set to exclude the effects of translocation. Note that the analyses of this paper or (*25*) do not exclude the possibility that the models might be able to do some extrapolation. Results of (*25*) show a lack of substantial extrapolation (from category 2 to 5 TCs), while any potential extrapolation in the current study could have been dwarfed by spectral bias. Regardless, innovations in architecture and training algorithms might be needed to further improve the ability of these models in learning physics, to the degree that extrapolation might become possible.

In conclusion, rapidly advancing AI weather models already demonstrate skill in forecasting unprecedented events such as the 2024 Dubai rainfall. Advancing our understanding of the mechanisms underlying their successes and limitations and disentangling the sources of their errors can help unlock the full potential of AI-driven weather and climate models for both accurate prediction and scientific discovery.

## MATERIALS AND METHODS

### AI weather models and ERA5 data

Three publicly available, trained AI weather models, GraphCast (*3*), AIFS (*5*), and FuXi (*4*), directly forecasted precipitation. GraphCast has multiple versions with different numbers of vertical levels; in this study, we use a 37-level configuration to generate 10-day forecasts at 0.25° spatial resolution from 6-hourly autoregressive rollouts. The model was trained on ERA5 reanalysis data from 1979 to 2017. AIFS was trained with ERA5 data from 1979 to 2020 and then fine-tuned on operational real-time IFS NWP analyses. FuXi was also trained with ERA5 reanalysis data (1979–2015 for training and 2016–2017 for validation) to generate 6-hourly forecasts up to 15 days ahead at a spatial resolution of 0.25°. A key difference of FuXi is that it uses a cascade of models trained for three sequential forecast periods of 0 to 5 days, 5 to 10 days, and 10 to 15 days, respectively, to optimize its performance for short- and medium-range weather forecasting. We also used Pangu-Weather and FourCastNet-V2 for circulation forecasts (see fig. S3).

The ERA5 reanalysis dataset (*86*) is used as ground truth to test the AI weather models in this study. The ERA5, in general, may not capture the highest observed precipitation totals (*47*). However, since AI models are trained with ERA5 data, we are testing the models in the world of ERA5 to better understand their potential and limitations.

### Identifying dynamically similar events using Lagrangian tracking

The Lagrangian detection and tracking algorithm described in Crawford *et al.* (*87*) is used to track extratropical cyclones in the ERA5 dataset. This algorithm identifies and tracks local minima in mslp, where “local” is defined within the 24 nearest neighbors (i.e., within a 5 × 5 box). The mslp fields are analyzed at a spatial resolution of 50 km and a temporal resolution of 3 hours.

To ensure that tropical storms are excluded from the analysis, TCs in the International Best Track Archive for Climate Stewardship (IBTrACS) dataset are compared with the cyclone centers identified by the tracking algorithm. If a TC from IBTrACS is located within 500 km of a tracked event, then that event is considered to be influenced by the TC and is excluded from the “Tropical cyclones removed” histogram of Fig. 2B.

After removing events influenced by TCs, dynamically similar events are then defined as those occurring during the boreal winter (November to April) and associated with a maximum precipitation rate within 1500 km of the cyclone center exceeding 60 mm per 12-hour period. The histogram of precipitation rates for “Dynamically similar events” is shown in Fig. 2B. Note that a single selected event can contribute multiple data points to the histogram, corresponding to precipitation rates ranging from 1 to 100 mm per 12-hour period.

### Return-period estimation

To quantify the rarity of the Dubai event’s rainfall, we estimate return periods using a peaks-over-threshold approach with a generalized Pareto distribution (GPD), which uses all large exceedances rather than only annual maxima (*88*). The variable of interest is the daily accumulated ERA5 rainfall, computed every 6 hours. For Dubai, we take the spatial maximum of this quantity within the 100 km–by–100 km box around Dubai. Exceedances above the 99th percentile of each series are declustered using a 24-hour window (retaining the cluster maximum) to reduce serial dependence. The excesses are then fitted with a GPD by maximum likelihood.

Let *u* denote the threshold, $\lambda_{u}$ the annual rate of declustered exceedances, and ξ and σ the fitted GPD shape and scale parameters, respectively. The return level for return period *T* is

$$z_{T}=\left\{\begin{array}{cc} u+\frac{\sigma }{\xi }\left[{\left(\lambda_{u}T\right)}^{\xi }-1\right], & \xi \neq 0\\ u+\sigma \text{log}\left(\lambda_{u}T\right), & \xi =0 \end{array}\right.$$ (1)

and, conversely, the return period of an event of magnitude $z^{*}>u$ is

$$T\left(z^{*}\right)=\left\{\begin{array}{cc} \frac{1}{\lambda_{u}}{\left[1+\xi \;\frac{z^{*}-u}{\sigma }\right]}^{1/\xi }, & \xi \neq 0\\ \frac{1}{\lambda_{u}}\text{exp}\left(\frac{z^{*}-u}{\sigma }\right), & \xi =0 \end{array}\right.$$ (2)

Uncertainty is estimated by bootstrap resampling of years with replacement and refitting the GPD. For the Dubai region, the April 2024 event has a median return period of 154 years (90% bootstrap confidence interval: 38 to 1.4 × 10^5^ years). This is overall consistent with the ∼333-year present climate estimate for this event in Francis *et al.* (*89*) based on Global Precipitation Measurement (GPM) rainfall and Coupled Model Intercomparison Project data in a climate attribution framework.

### Fine-tuning GraphCast

To develop a causal experiment for translocation, we fine-tuned GraphCast using a modified training dataset. In this dataset, precipitation values for dynamically similar events (defined above) outside the Dubai region (a 100 km–by–100 km box centered at Dubai) are capped at 15 mm per 6-hour period (30 mm per 12-hour period). We restrict the fine-tuning period to 2008 to 2017 for computational feasibility. We also adjusted the training rollout period from the original 3 days to 1 day to accommodate memory constraints {[the impact of this is minimal, as shown by the global anomaly correlation coefficient (ACC) and root mean square error (RMSE) result in Fig. 5 in (*54*)]}. For the fine-tuning, a learning rate of 1 × 10^−6^ and five epochs are used. The machinery provided by Subich (*54*) made fine-tuning GraphCast possible. Fine-tuning AIFS, which is a newer model, has not been straightforward and is left for future studies.

### Calculation of ERF

To quantify the extent to which the neural network of an AI weather model uses spatially nonlocal information for the prediction of a variable at a given location, we use the ERF, which can be numerically calculated for any architecture (*51*, *52*). The ERF measures how much a specific output is influenced by small changes in the input, identifying the regions of the input that are more influential. Calculating the ERF involves backpropagating a signal from the output layer to the input layer, similar to techniques such as saliency maps (*90*) and layer-wise relevance propagation (*91*), which have been recognized as effective visualization tools in climate applications (*92*–*94*).

To calculate the ERFs of AIFS and GraphCast for predicting precipitation over Dubai for a single time step (6 hours), the models are initialized at 12:00 UTC on 15 April 2024. Within the Dubai region (defined as 23°N to 28°N latitude and 50°E to 60°E longitude), we identify the grid cell with the strongest precipitation for the next forecast at time *t*, $P_{\text{Dubai}}=P\left(y=\text{Dubai},t\right)$. *y* represents location (latitude, longitude, and altitude). Note that P(y,t) is one of the variables in the state vector X(y,t), the input and output of the models’ neural network NN

X(y,t+1)=NN[X(y,t−1), X(y,t), θ] (3)

where θ represents the weights of the neural network. We compute the partial derivative $\partial P_{\text{Dubai}}/\partial X$. This partial derivative is calculated by backpropagating a gradient from the output to the input of NN, similar to the standard process of propagating the error gradient relative to a loss function (*51*). Following previous studies (*51*), the gradient is set to zero for all outputs except the specific point of interest, $P_{\text{Dubai}}$, allowing the ERF to reveal the sensitivity of this output to variations in the input.
