# Supplementary material for: Predicting regional gray swans via translocation: AI weather models and Dubai’s unprecedented 2024 rainfall
Source: Sci Adv. 2026 Oct 7;12(41):eady9406. doi: 10.1126/sciadv.ady9406 (PMC13644771; doi:10.1126/sciadv.ady9406)
Supplement: Supplementary file 1 [file sciadv.ady9406_sm.pdf]

Science Advances
AAAS

Supplementary Materials for

Predicting regional gray swans via translocation: AI weather models and Dubai's unprecedented 2024 rainfall

Y. Qiang Sun *et al.*

Corresponding author: Y. Qiang Sun, qiangsun@nju.edu.cn; Pedram Hassanzadeh, pedramh@uchicago.edu

Sci. Adv. **12**, eady9406 (2026)
DOI: 10.1126/sciadv.ady9406

This PDF file includes:

Figs. S1 to S6

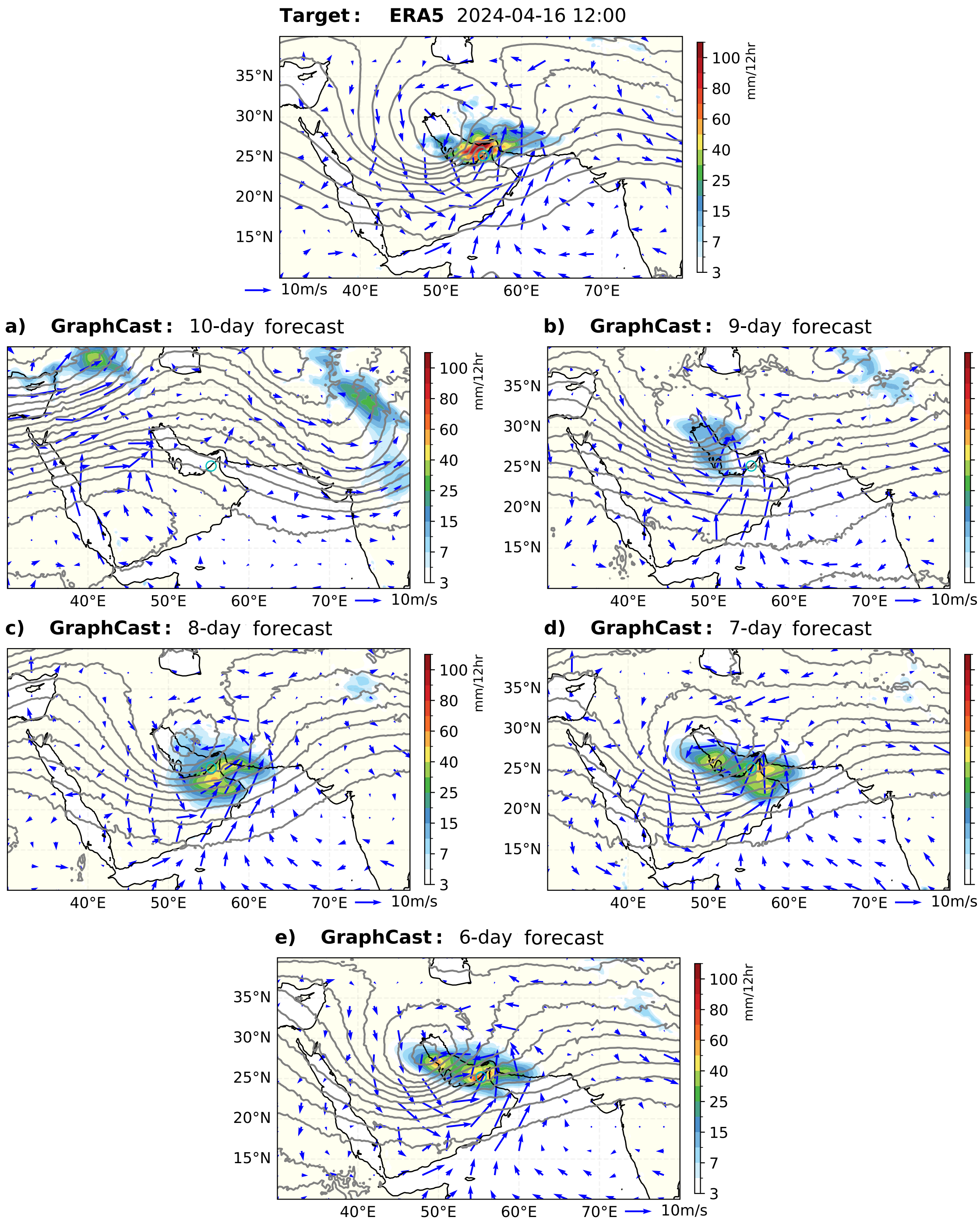

Figure S1: **GraphCast forecasts of the Dubai event at various lead times.** **(a)** 10 days; **(b)** 9 days; **(c)** 8 days lead time, note the correct location and the precipitation amplitudes that exceed the training maximum near Dubai already; **(d)** 7 days; **(e)** 6 days.

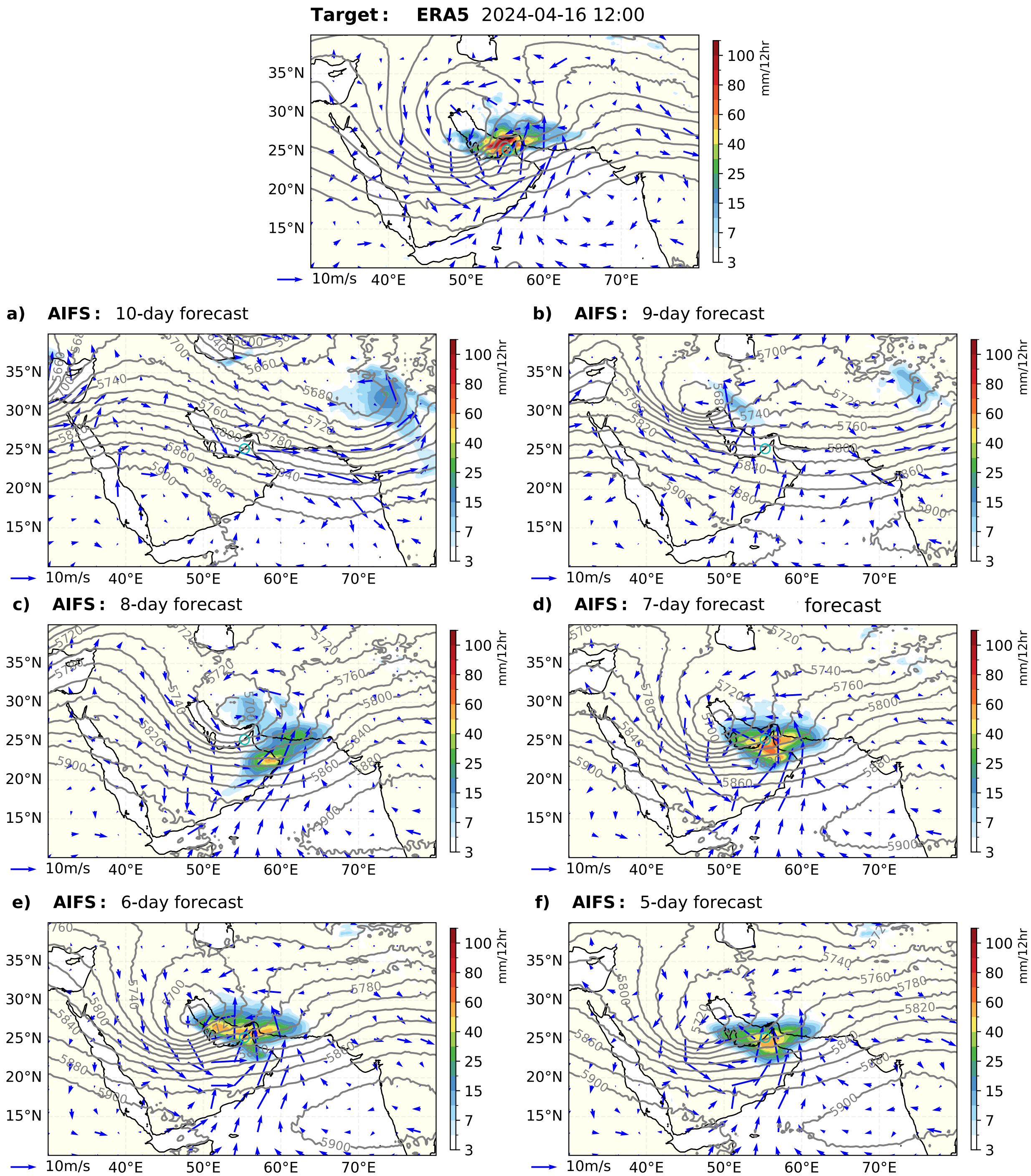

Figure S2: **AIFS forecasts of the Dubai event at various lead times.** **(a)** 10 days; **(b)** 9 days; **(c)** 8 days; **(d)** 7 days; **(e)** 6 days; **(f)** 5 days.

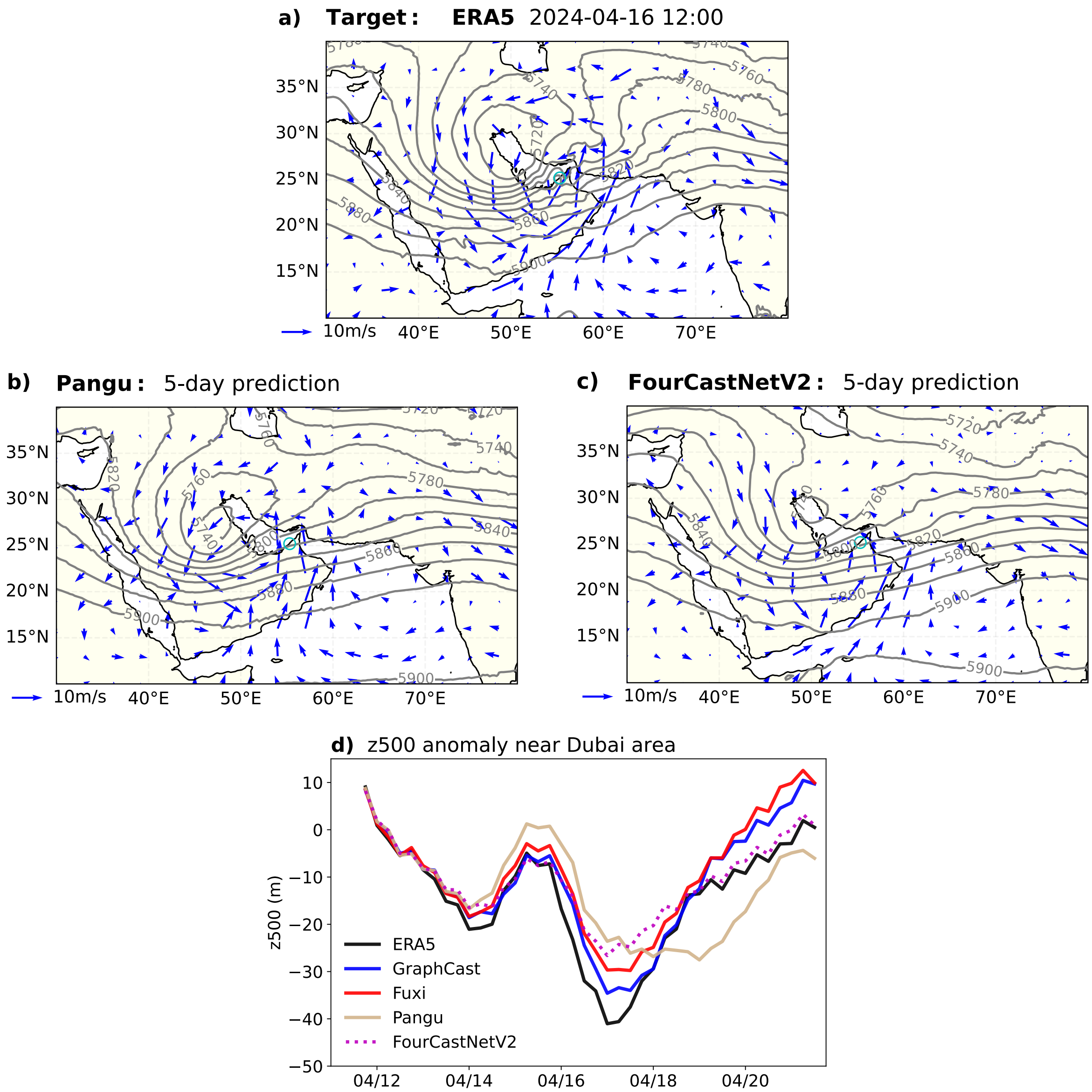

Figure S3: **Forecasts by the Pangu-Weather and FourCastNetV2 models for the event. (a)** Geopotential height at 500 hPa (z500) and 850 hPa wind vector from ERA5 reanalysis; **(b)** Forecast from the Pangu-Weather model, initialized on April 11th; **(c)** Forecast from the FourCastNetV2 model, initialized on April 11th; **(d)** Time evolution of the area-averaged geopotential height over the Dubai region as represented in ERA5 and in forecasts produced by the four AI models in this study.

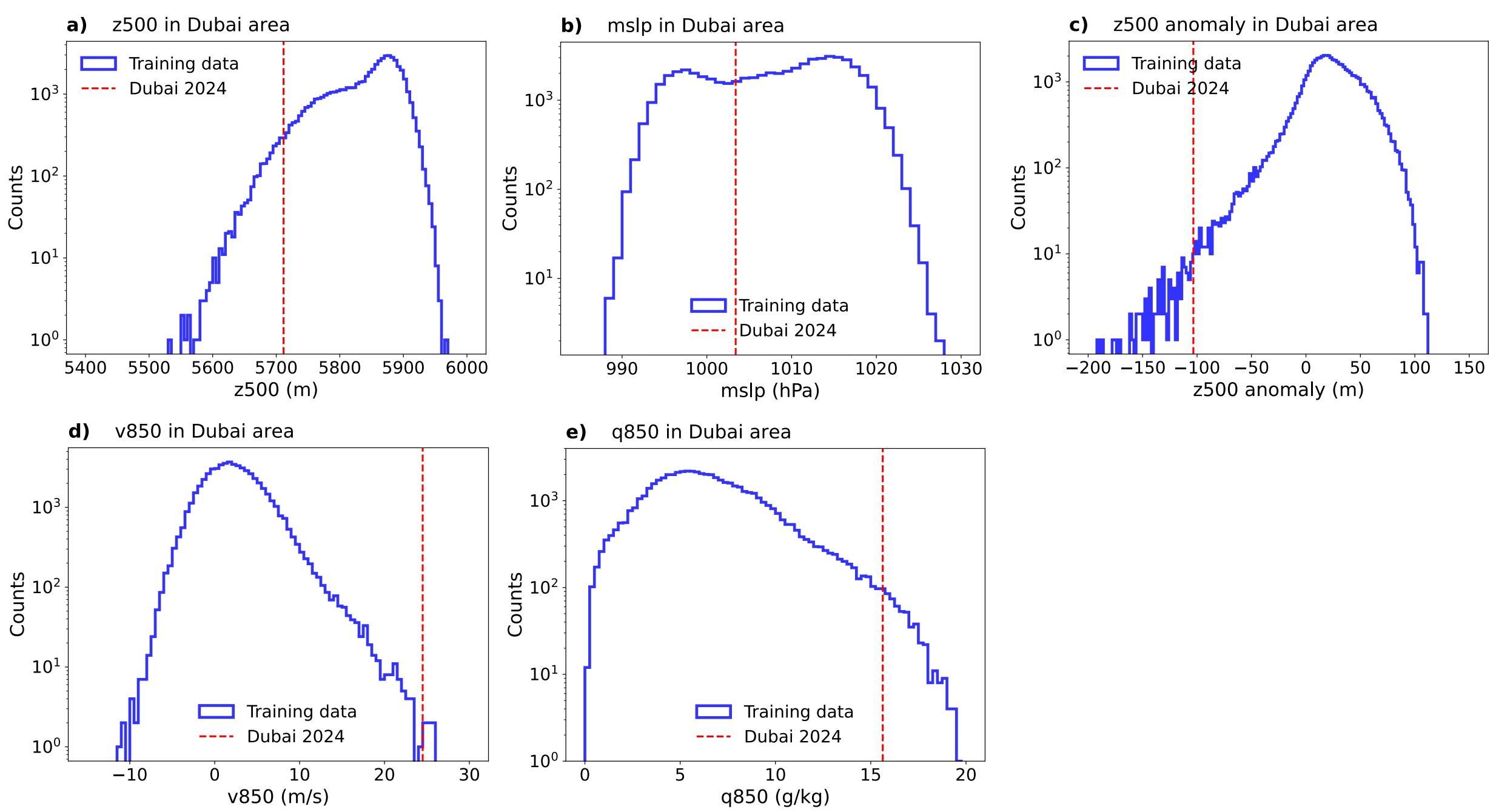

Figure S4: **Rarity of the Dubai event in different variables.** **(a)** Geopotential height at 500 hPa (z500); **(b)** Mean sea level pressure (mslp); **(c)** Geopotential height anomaly at 500 hPa (z500 anomaly); **(d)** Meridional wind at 850 hPa (v850); **(e)** Specific humidity at 850 hPa (q850). When examined individually, none of these variables is OOD. Nonetheless, when considered together, some of the joint distributions, most notably that of v850-q850, indicate unprecedented meridional moisture transport (Figure 2D in the main text).

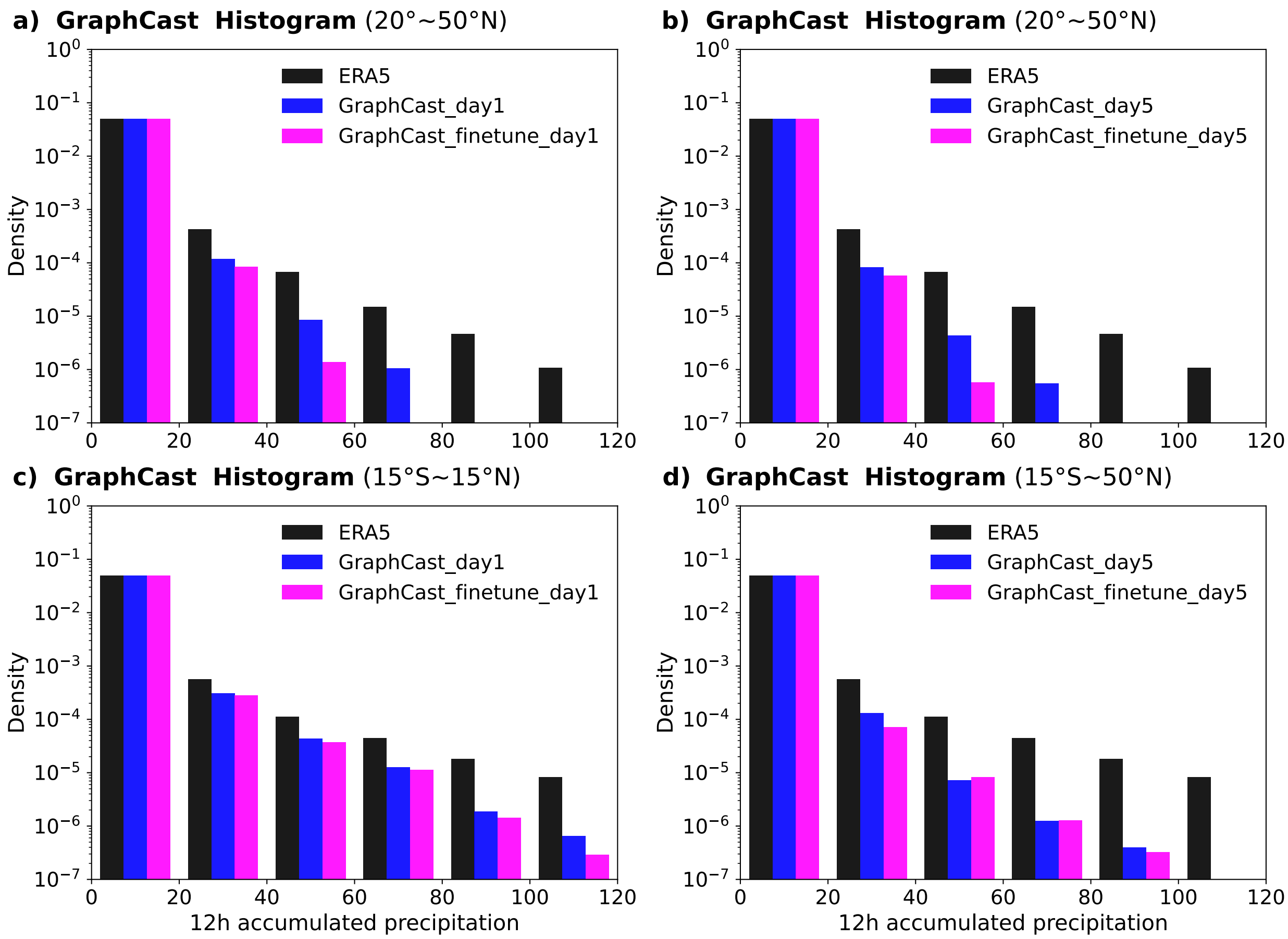

Figure S5: **Differences in precipitation histograms between the original GraphCast and the fine-tuned version after capping rainfall in dynamically similar events (mid-latitude storms).** **(a–b)** Northern Hemisphere midlatitudes (20°N–50°N); **(c–d)** Tropics (15°S–15°N). Histograms include all events, not just dynamically similar ones. The precipitation capping of the Northern Hemisphere midlatitude cyclone has a substantially large impact on the Northern Hemisphere mid-latitudes but limited effects on the tropics, where the extreme precipitation has different dynamics.

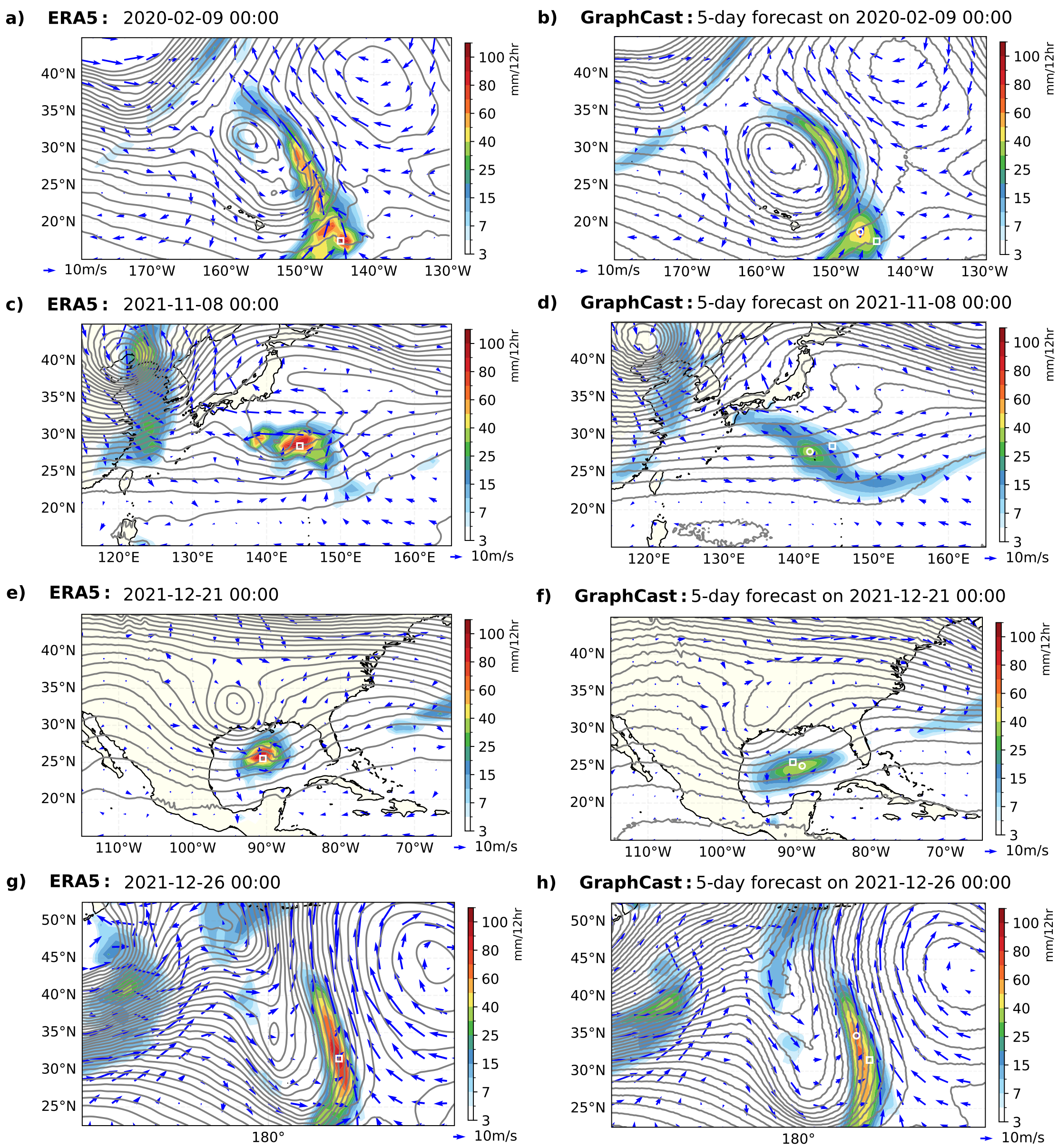

Figure S6: **GraphCast forecasts of global extreme events.** Similar to Figure 6 in the main text, this figure presents four additional cases. The left column shows ERA5 reanalysis data for extreme rainfall events, while the right column displays GraphCast's 5-day forecasts for the same fields. In all cases, the predicted precipitation is significantly weaker than the observed values.
